# Proteomics of human platelet lysates and insight from animal studies on platelet protein diffusion to hippocampus upon intranasal administration

**DOI:** 10.1063/5.0196553

**Published:** 2024-05-06

**Authors:** Nhi Thao Ngoc Le, Chia-Li Han, Liling Delila, Ouada Nebie, Hsin-Tung Chien, Yu-Wen Wu, Luc Buée, David Blum, Thierry Burnouf

**Affiliations:** 1 International Ph.D. Program in Biomedical Engineering, College of Biomedical Engineering, Taipei, Taiwan; 2 Master Program in Clinical Genomics and Proteomics, College of Pharmacy, Taipei Medical University, Taipei, Taiwan; 3 Graduate Institute of Biomedical Materials and Tissue Engineering, College of Biomedical Engineering, Taipei, Taiwan; 4 Université Lille, Inserm, CHU-Lille, U1172, Lille Neuroscience and Cognition, Lille, France; 5 Alzheimer and Tauopathies, Labex DISTALZ, Lille, France; 6 NeuroTMULille, Lille Neuroscience and Cognition, Lille, France; 7 NeuroTMULille, Taipei Medical University, Taipei, Taiwan; 8 International PhD Program in Cell Therapy and Regeneration Medicine, Taipei Medical University, Taipei, Taiwan; 9 Ph.D. Program in Graduate Institute of Mind Brain and Consciousness, College of Humanities and Social Sciences, Taipei Medical University, Taipei, Taiwan

## Abstract

Human platelet lysates (HPLs) from allogeneic platelet concentrates (PCs) are biomaterials, which are rich in various trophic factors, increasingly used in regenerative medicine and biotherapy. Understanding how preparation methods influence the HPL protein profile, biological function, and clinical outcomes is crucial. Our study sheds light on the proteomes and functionality of different HPLs, with the aim of advancing their scientifically grounded clinical applications. To achieve this, PCs suspended in plasma underwent three distinct processing methods, resulting in seven HPL types. We used three characterization techniques: label-free proteomics and tandem mass tag (TMT)-based quantitative proteomics, both before and after the immunodepletion of abundant plasma proteins. Bioinformatic tools assessed the proteome, and western blotting validated our quantitative proteomics data. Subsequent pre-clinical studies with fluorescent labeling and label-free proteomics were used as a proof of concept for brain diffusion. Our findings revealed 1441 proteins detected using the label-free method, 952 proteins from the TMT experiment before and after depletion, and 1114 proteins from the subsequent TMT experiment on depleted HPLs. Most detected proteins were cytoplasmic, playing key roles in catalysis, hemostasis, and immune responses. Notably, the processing methodologies significantly influenced HPL compositions, their canonical pathways, and, consequently, their functionality. Each HPL exhibited specific abundant proteins, providing valuable insight for tailored clinical applications. Immunoblotting results for selected proteins corroborated our quantitative proteomics data. The diffusion and differential effects to the hippocampus of a neuroprotective HPL administered intranasally to mice were demonstrated. This proteomics study advances our understanding of HPLs, suggesting ways to standardize and customize their production for better clinical efficacy in regenerative medicine and biotherapy. Proteomic analyses also offered objective evidence that HPPL, upon intranasal delivery, not only effectively diffuses to the hippocampus but also alters protein expression in mice, bolstering its potential as a treatment for memory impairments.

## I. INTRODUCTION

Human platelet lysates (HPLs) are potent biomaterials, which are rich in multiple trophic factors derived from the lysis, activation, or degranulation of platelets.^1,2^ HPLs are increasingly sourced from clinical-grade allogeneic platelet concentrates (PCs) produced by licensed blood establishments.^3^ Over time, HPLs have been recognized as invaluable cell culture supplements in the cell therapy industry for xeno-free propagation of therapeutic mesenchymal stromal cells (MSCs), differentiated cells, like chondrocytes, and immune cells, including CAR-T cells.^4–6^ In addition, HPLs are progressively adopted across a spectrum of regenerative medicine applications,^3^ such as for treating osteoarthritis^7,8^ and musculoskeletal injuries,^9^ stimulating bone regeneration,^10^ releasing dry eye syndrome,^11,12^ and curing diabetic ulcers.^13^ Additionally, tailor-made HPLs, enriched in neurotrophic factors, neurotransmitters, antioxidants, and protease inhibitors, are showing promise in preclinical studies as a pleiotropic biotherapy of complex neurological disorders like Parkinson's disease,^14,15^ Alzheimer's disease,^16^ amyotrophic lateral sclerosis,^17^ traumatic brain injury,^18,19^ and stroke,^20^ as reviewed recently.^21^

In view of these translational interests, a consistent, scientifically rooted understanding of how the protein composition of HPLs influences their clinical safety and efficacy is required to substantiate pre-clinical and clinical developments. This is further justified as there exist variables in HPL preparation, encompassing individual blood donor variability, methods of PC collection, pooling, and platelet lysis, degranulation, and further processing.^3,22,23^ Such manufacturing differences are thought to yield HPLs with distinct protein profiles, potentially altering their effectiveness in cell expansion and tissue regeneration and protection, as observed when using autologous platelet rich plasma.^24,25^ For instance, HPLs can be prepared through direct freeze-thawing of PCs suspended in plasma or from isolated platelets.^14,26,27^ Some undergo calcium salt-triggered seroconversion,^26^ while others are heat-treated^14,28^ or subjected to virus-reduction treatments,^19,26,29^ each with its potential effects on protein content and function. Given these variabilities, conducting comprehensive proteomic analyses using advanced techniques of label-free and tandem mass tag (TMT)-labeled mass spectrometry (MS) can be of a significant scientific value to objectively guide the translational applications of HPLs. Such analyses can inform the standardization of HPLs production,^30^ support scientifically based development, and enhance optimal clinical outcomes.

This study aims to (1) utilize proteomics for a comprehensive analysis of the proteome and key protein cohorts in seven types of HPLs; (2) investigate how different processing techniques affect the protein compositions and safety profiles of HPL; (3) explore the variations in these proteins and their associated biological pathways, aligning them with relevant clinical applications; and (4) demonstrate the use of proteomics, supported by fluorescent protein labeling, to understand the effectiveness of a neuroprotective HPL, administered intranasally, in reaching the hippocampus of mice, as part of its potential clinical application in treating neurological disorders.^21^ The proteomes identified in this study seek to bridge the existing scientific gap, aiding in the standardization and optimal use of HPLs. Our findings should, thus, provide insight crucial for producing safe, effective, and personalized HPL-based treatments in regenerative medicine, steering the field toward much-needed more nuanced and tailored clinical applications.

## II. RESULTS

In our investigation, we generated seven unique HPLs using clinical-grade apheresis PCs suspended in plasma and sourced from healthy blood donors as is the current trends for applications in cell therapy and regenerative medicine. These HPLs and their preparation methods are summarized in Table I.

**TABLE I.** Human platelet lysates (HPLs) evaluated: preparation modes and their goals. Platelet concentrate (PC); Freeze-thawed platelet lysate (FTPL); serum-converted platelet lysate (SCPL); heat-treated, serum-converted platelet lysate (HSCPL); platelet pellet lysate (PPL); heat-treated platelet pellet lysate (HPPL); HPPL subjected to 0.2–0.1-*μ*m microfiltration (MHPPL); and 19-nm nano-filtered HPPL (NHPPL).

Name of HPL	Mode of preparation	Intended goal(s) for the preparation modes
FTPL	• Freeze (−80 °C)—Thaw (37 °C) of PC	• Mechanically break the cell membrane to release platelet contents
SCPL	• Calcium chloride treatment of PC	• Chemically activate platelets to release cellular contents
HSCPL	• Heat-treatment of SCPL (56 °C, 30 min)	• Reduce fibrinogen, thrombin generation activity, inactivate proteolytic and pro-thrombotic enzymes
PPL	• Centrifugation of PC to isolate platelets	• Removes the plasma compartment
	• Freeze (−80 °C)—Thaw (37 °C) of platelets	• Mechanically breaks the platelet membranes to release platelet contents
HPPL	• Heat-treatment of PPL (56 °C, 30 min)	• Reduces fibrinogen, thrombin generation activity, inactivate proteolytic and pro-thrombotic enzymes
Filtered HPPLs	• Micro- and 19-nm nano-filtration of HPPL	• Removes enveloped and non-enveloped viruses

### A. Specification of identified proteomes

#### 1. Protein contents

**FIG. 1.**
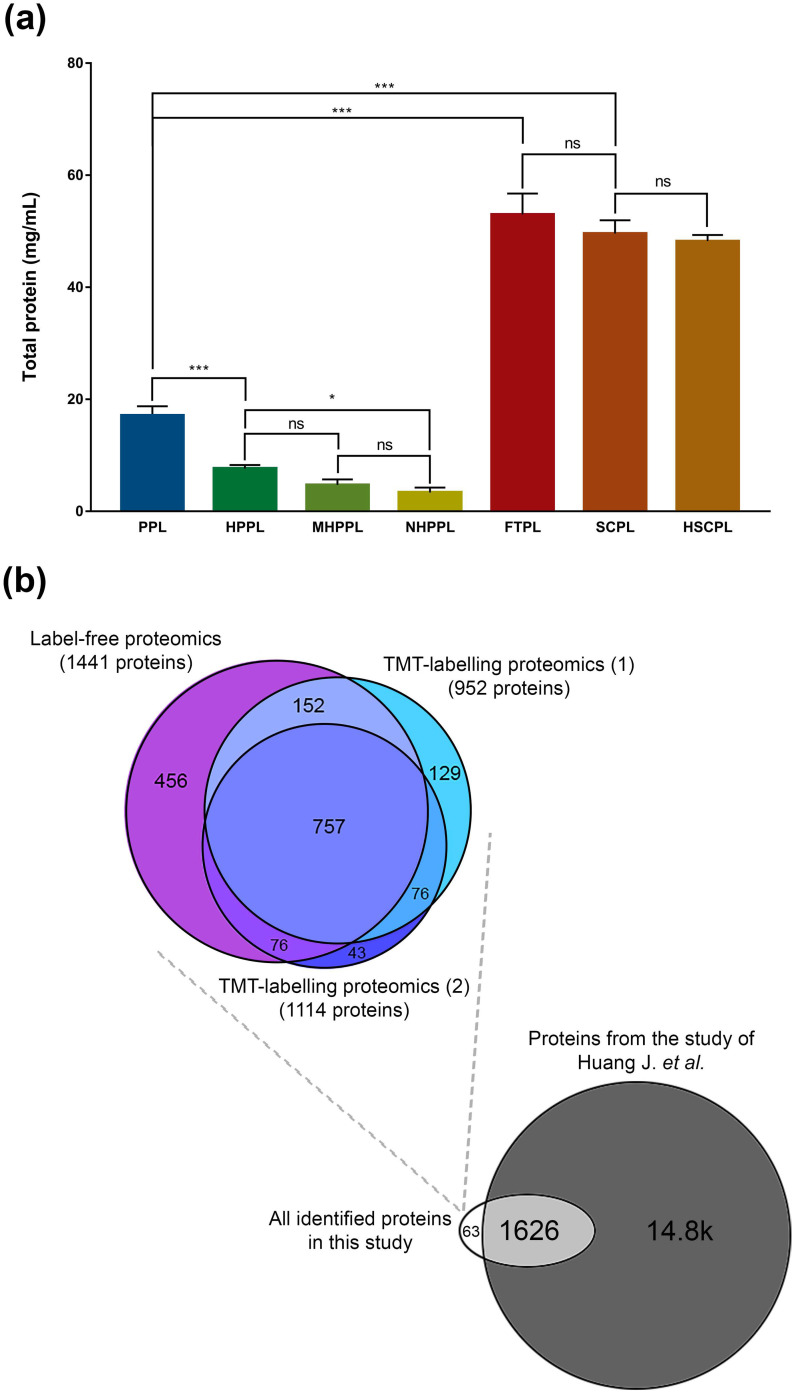
(a) Total proteins (mg/ml) in human platelet lysates (HPLs). Results are expressed as the mean ± SD (N = 4, biological replicates). (^*^p < 0.05, ^***^p < 0.001) ns, not significant; (b) Venn diagram of the total number of identified proteins using label-free vs those from the TMT-labeled techniques; and from the current study using a label-free technique vs a study by Huang *et al.*^32^

The BCA protein dosage revealed that the total protein content is, as expected, significantly higher (*p* < 0.001) in FTPL, SCPL, and HSCPL, up to 2.5-times more than in PPL and its derivatives [Fig. 1(a)]. The protein content in HPPL dropped significantly (*p* < 0.001) post the 56 °C/30 min treatment, whereas only a minor non-significant shift (*p* > 0.05) was observed in HSCPL post the same treatment. The nanofiltration step led to a slight reduction of protein content in HPPL.

Using label-free proteomics, we identified a diverse set of 1441 proteins across the HPLs (supplementary material 1, Table S1). Different numbers of proteins: 1296 (PPLs), 1117 (HPPL), 1011 (MHPPL), 897 (NHPPL), 592 (FTPL), 512 proteins (SCPL), and 509 (HSCPL) were detected in individual HPLs. To gain a more nuanced understanding of protein abundances, we also undertook two TMT-labeled experiments (supplementary material 1, Tables S2 and S3). The initial experiment, termed “TMT-labeled proteomics (1),” identified 952 proteins across five HPLs of before and after plasma-abundant protein depletion. The second experiment, termed “TMT-labeled proteomics (2),” identified 1114 proteins across seven depleted HPLs (dHPLs).

Overall, the cumulative data from these three label-free or TMT proteomics investigations unveiled 1689 proteins that characterize HPLs, with 757 proteins being consistently present across the different HPLs types [Fig. 1(b)]. Interestingly, a comparative analysis with a recent platelet proteome study by Huang *et al.*^31^ showed a 96.3% overlap of 1626 proteins. Yet, our dataset uniquely identified 63 proteins not previously reported (supplementary material 3, Table S1).

#### 2. Protein annotations

**FIG. 2.**
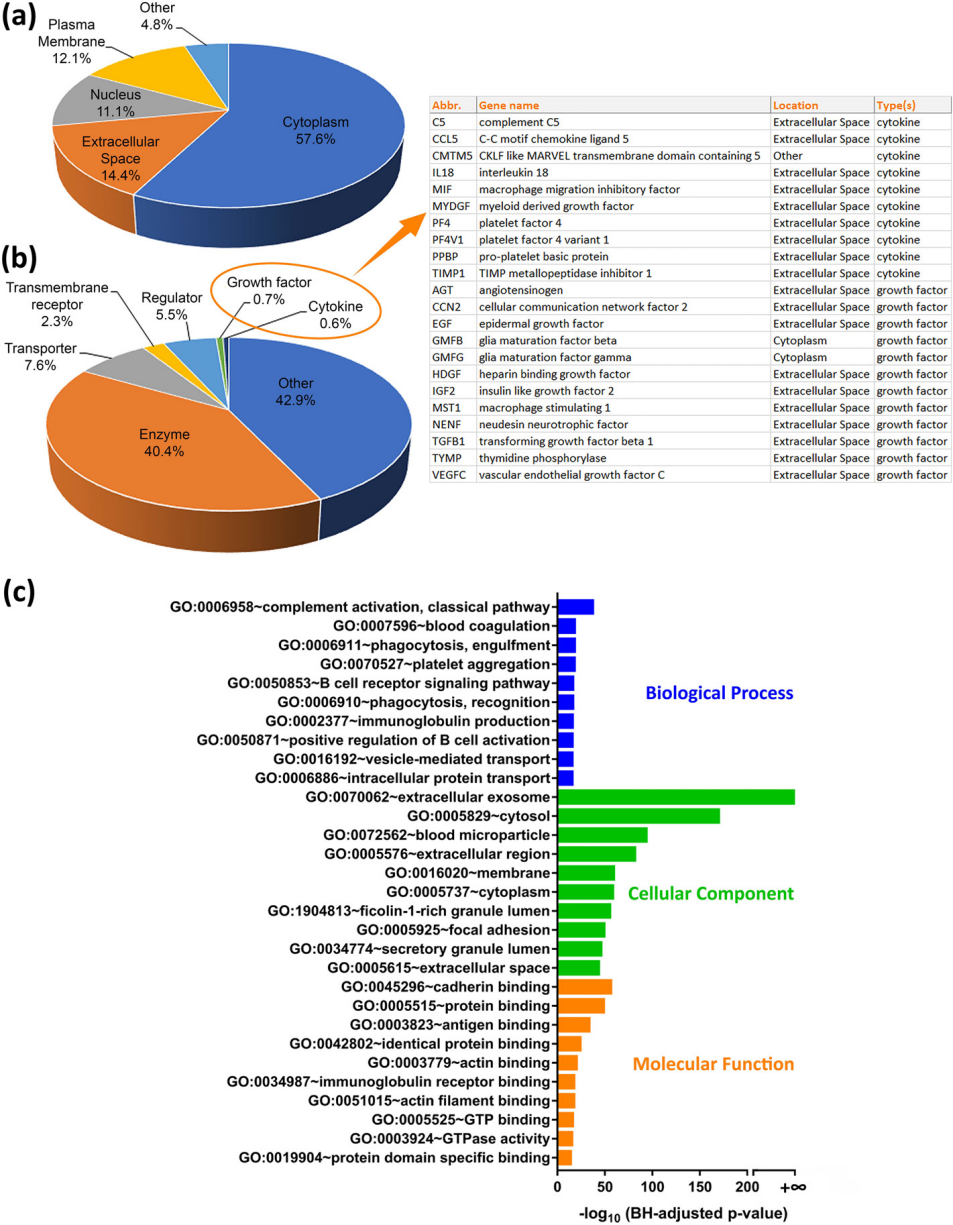
Categorization of human platelet lysates (HPLs) by (a) subcellular localization and (b) general function and a corresponding list of growth factors and cytokines. Data are presented as percentages of the total number of identified proteins in a given group per total number of proteins recognized by the IPA. (c) Categorization of HPLs by gene ontology (GO)-biological processes, GO-cellular components, and GO-molecular functions using DAVID. Data are presented as significance −log10 (BH-adjusted p value) of the prediction term.

To contextualize our findings, we employed an Ingenuity Pathway Analysis (IPA) to examine the subcellular localization [Fig. 2(a)] and functionality [Fig. 2(b)] of the 1689 identified proteins. A significant proportion (57.6%) was linked with the cytoplasm followed by those associated with extracellular space (14.4%), plasma membranes (12.1%), and nuclei (11.1%). Functionally, these proteins were playing roles associated with catalytic activities (40.4%), transport (7.6%), regulation (5.5%), and signaling (2.3%). Among them, 22 growth factors and cytokines, including platelet factor 4, vascular endothelial growth factor, myeloid-derived growth factor, and C-X-C motif chemokine 7, etc., were detected. Delving deeper, the HPLs proteins were found to be central to vital biological processes like complement activation, blood coagulation, and phagocytosis, while a majority contributed to protein-binding activities [Fig. 2(c)]. Moreover, almost 90% of the identified proteins were linked to exosomes—or extracellular vesicles—(as evidenced by ExoCarta and Vesiclepedia databases, Fig. S2, supplementary material 2).

### B. TMT-labeled proteomics characterization of HPLs

#### 1. Proteome variations

**FIG. 3.**
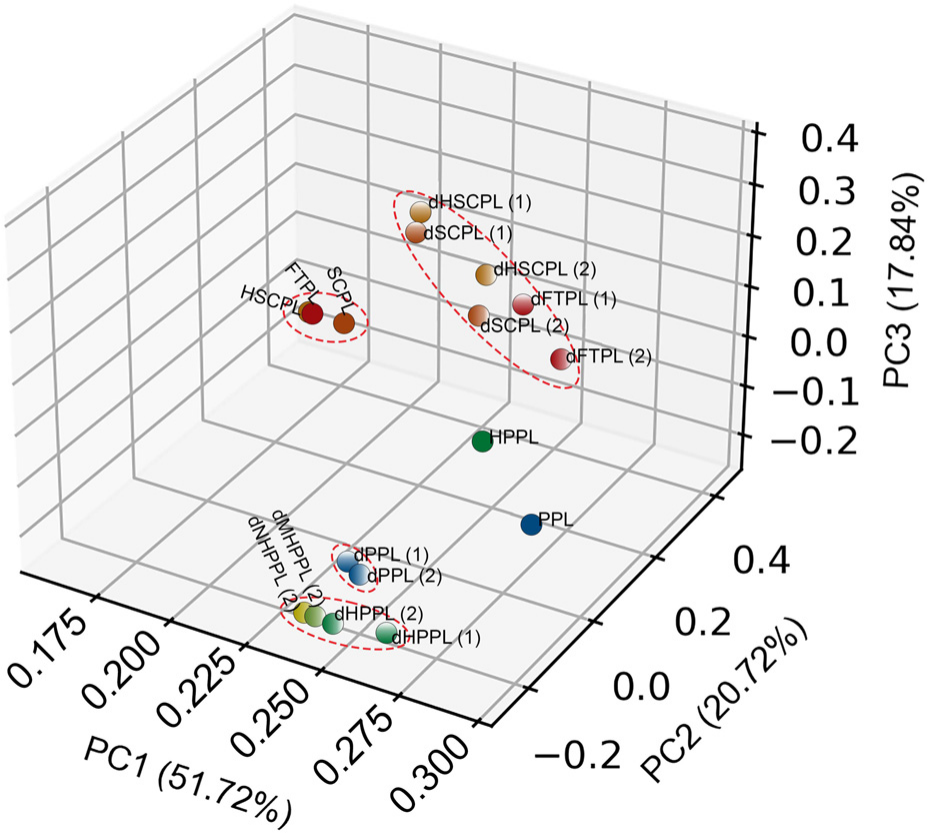
Principal component analysis (PCA) plot showing a grouping of different human platelet lysates (HPLs) based on their protein expressions.

Using PCA, we analyzed variations in proteomes among HPLs in the two TMT-labeled experiments (Fig. 3). We identified two distinctive protein clusters: one pre-depletion and another post-depletion. Furthermore, dHPLs in both TMT-labeled experiments were similar and grouped the same way as HPLs, reflecting their shared proteomic changes in response to depletion. FTPL, SCPL, and HSCPL show considerable similarities. In contrast PPL and HPPLs, including HPPL, MHPPL, and NHPPL, are distinct. Interestingly, we observed a trend toward a greater number of quantifiable proteins in dHPLs (Fig. S3, supplementary material 2). Specifically, depletion introduced an exclusive set of 8–14 proteins per HPL, cumulatively adding 52 proteins to the overall profile (supplementary material 3, Table S2).

#### 2. Proteomic signature of HPLs

**FIG. 4.**
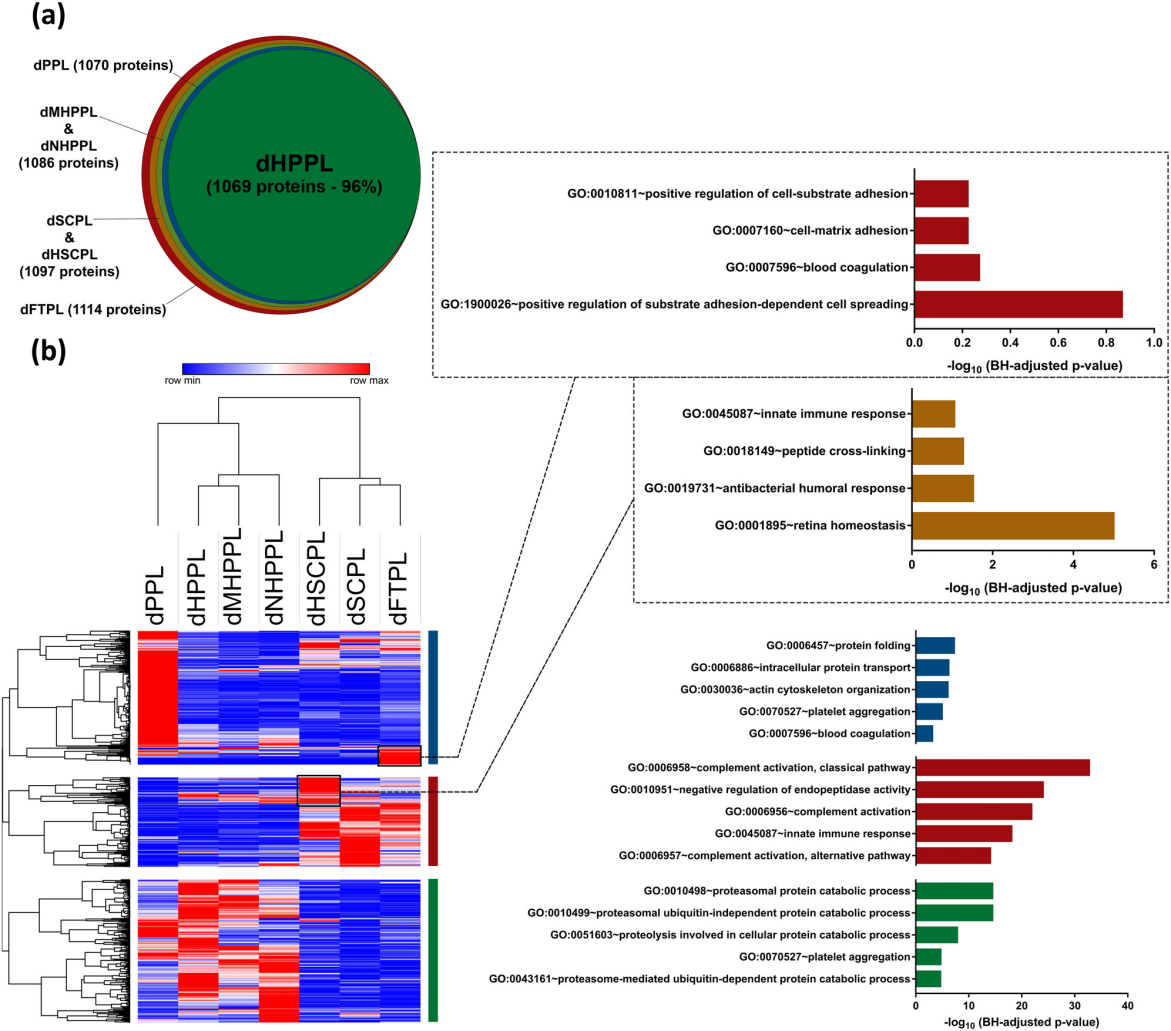
(a) Venn diagram of the number of quantified proteins in human platelet lysates (HPLs); and (b) heatmap of quantified protein in HPLs and categorization of their respective clusters by gene ontology-biological processes. Presented as significance −log10(BH-adjusted p value) of the prediction term.

Our TMT-based LC-MS/MS analysis of the seven dHPLs identified a set of 1114 proteins (with FDRs of < 1%). The Venn diagram [Fig. 4(a)] highlighted a commonality, with 96% of these proteins shared across all dHPLs, with dFTPL containing all of them. dSCPL and dHSCPL exhibited 1097 proteins that also covered 1086 proteins in the filtered-dHPPLs (MHPPL and NHPPL) and 1070 proteins in dPPL and dHPPL (supplementary material 3, Table S3).

Unsupervised hierarchical clustering, based on protein abundance, led to classify dHPLs proteomes into three groups: (1) dPPL, (2) dHPPLs (dHPPL-dMHPPL-dNHPPL), and (3) dFTPL-dSCPL-dHSCPL [Fig. 4(b)]. The Pearson correlation heatmap (Fig. S4, supplementary material 2) further illustrated their inter-relations. An analysis of the most abundant proteins in each group linked them to specific GO-biological processes [Fig. 4(b)]. For instance, proteins in dPPL influence protein folding, intracellular transport, cytoskeletal organization, and platelet aggregation, while those in dHPPLs impact protein catabolism. The dFTPL-dSCPL-dHSCPL group includes proteins involved in complement activation and innate immune responses. dFTPL proteins play roles in cell spreading, while dHSCPL proteins are vital for retina homeostasis (list of highly abundant proteins specific to HPLs is in supplementary material 3, Table S4).

#### 3. Assessing the impact of processing on dHPLs

**FIG. 5.**
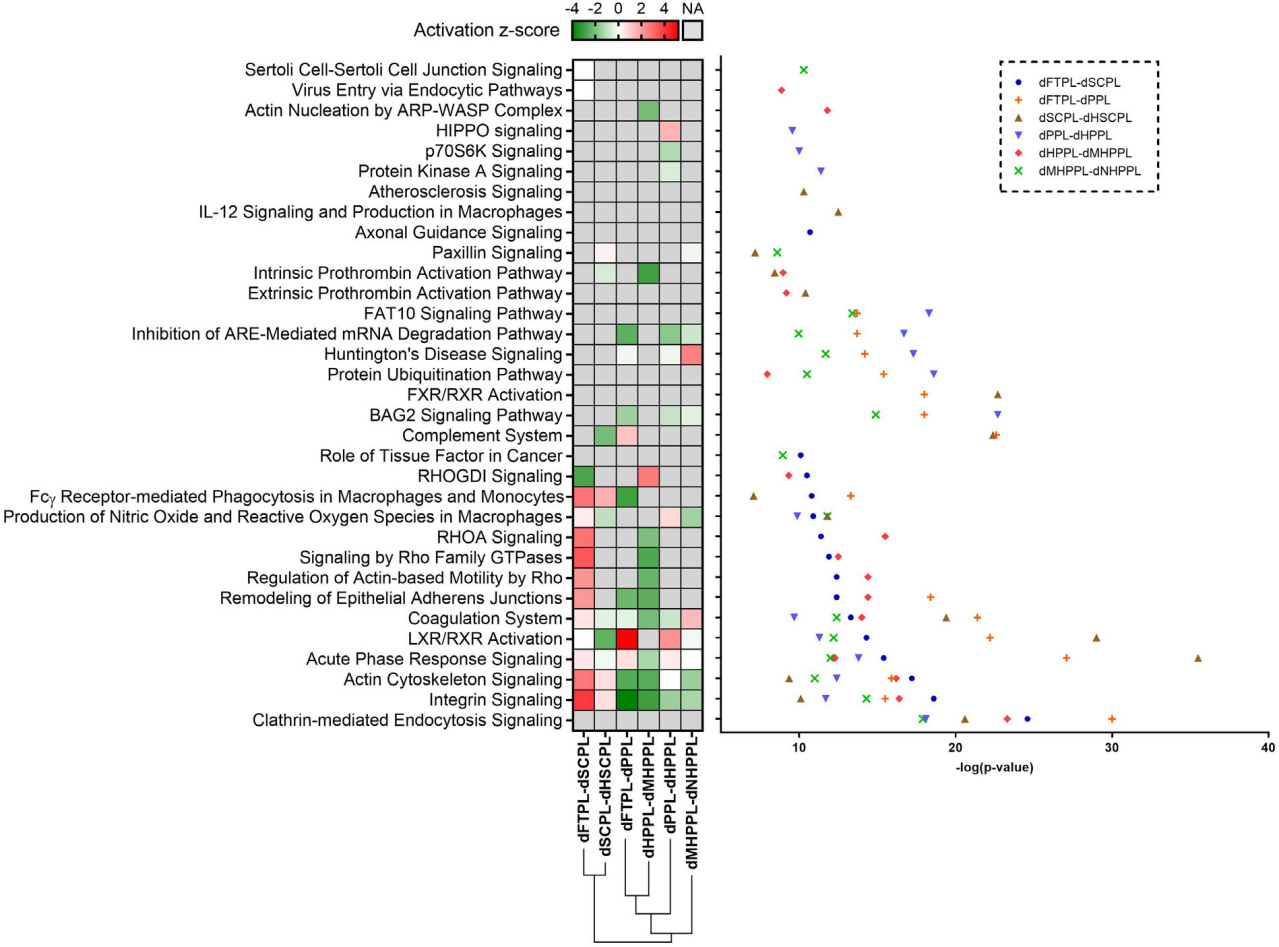
Major canonical pathways discovered by the Ingenuity pathway analysis (IPA) and the degrees of activation of proteins with significant changes in abundances at each processing step. The pathways are given in statistically significant (p value) and activation z-scores (a z-score of > 0 suggests activation, whereas a z-score of < 0 shows inhibition; NA, not available).

In our research, we used distinct processing steps to produce different HPL biomaterials. As expected, these procedures greatly influenced protein profiles compared to the standard FTPL lysates. Proteins whose abundances significantly changed compared to their parental dHPLs were further analyzed using IPA. A comparison of dHPLs before and after processing revealed pronounced shifts in relative protein abundances (Fig. S5, supplementary material 2). The most notable differences were tied to the complete removal of plasma, setting dPPL apart from dFTPL, and heat-treatment, setting dHPPL apart from dPPL. These changes impacted as many as 900 and 820 proteins, respectively. Recognizing the significance of these changes, we used IPA to explore associated canonical pathways. The protein shifts correlated with specific canonical metabolic pathways, as shown by unsupervised hierarchical analysis (Fig. 5). Clusters of changes emerged from the methods used to produce dSCPL and dPPL. Calcium activation led to an increase in signaling pathways associated with actin, cytoskeletal organization (RHOA signaling and Rho signaling), cell adherens signaling, and the endocytic inflammatory response (macrophages signaling and acute-phase response signaling). However, these pathways decreased in dFTPL-dPPL and dHPPL-dMHPPL processes. Post heat-treatment, dHSCPL showed a slight decrease in signals from the complement system. Additionally, changes in platelet intercellular signaling pathways (BAG2 signaling, inhibition of ARE-mediated mRNA degradation pathway, HIPPO signaling, and p70S6K signaling) were observed following the heat-treatment of dPPL and filtration of dHPPL.

### C. Validation through western blot analysis

**FIG. 6.**
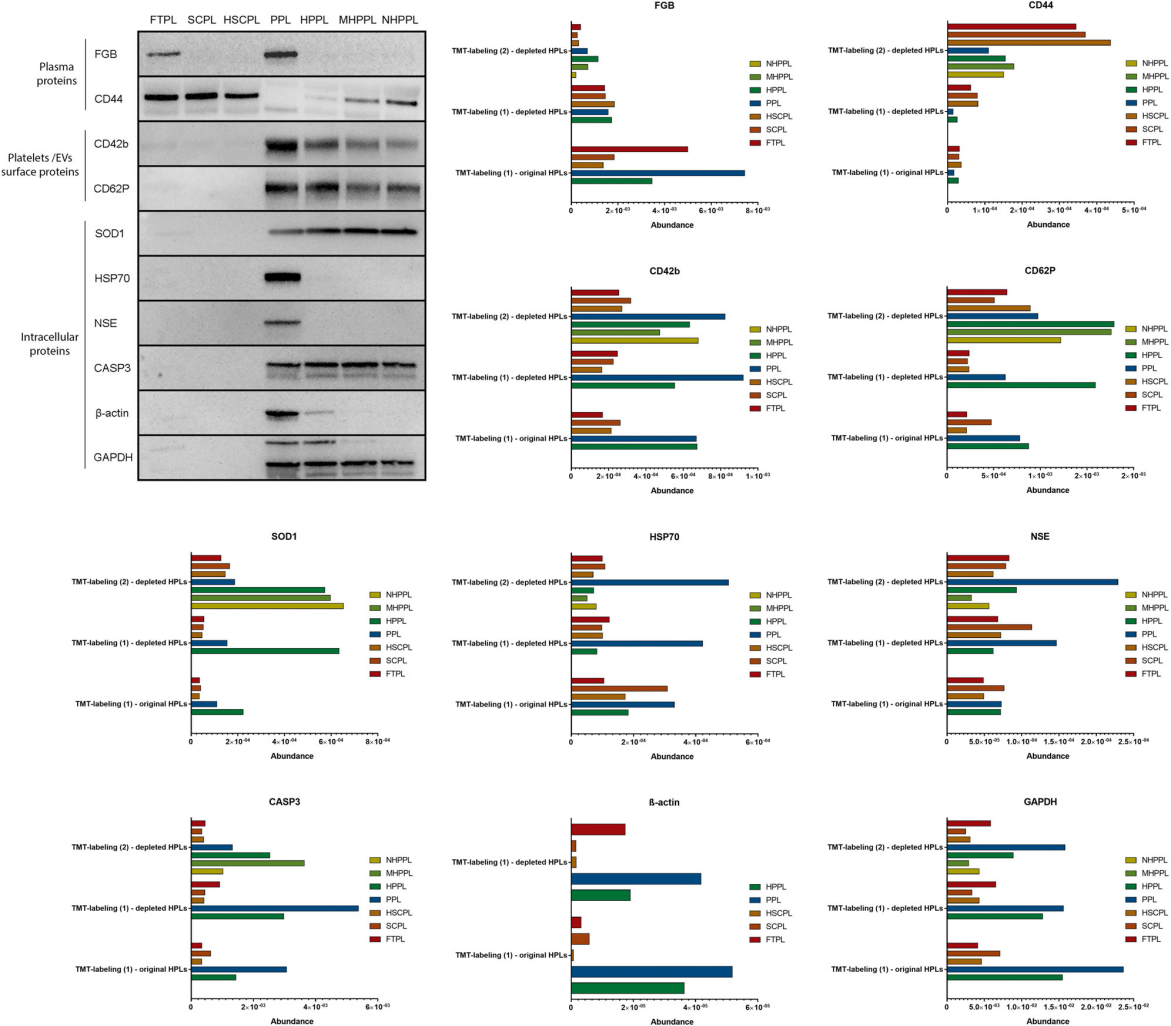
Western blot results of proteins in human platelet lysates (HPLs) and their respective quantification results from proteomics including original- and depleted-HPLs from TMT-proteomics (1) and depleted-HPLs from TMT-proteomics (2).

To corroborate the findings from our proteomics evaluation, we conducted western blot analyses. We targeted a range of proteins, including those from plasma, inside platelets, and present on platelet surfaces (Fig. 6). The proteins studied were FGB (P02675), CD44 (P16070), SOD1 (P00441), GAPDH (P04406), CD42b (P07359), NSE (P09104), HSP70 (P0DMV9), CD62P (P16109), CASP3 (P42574), and β-actin (P60709).

Our western blot tests robustly supported the validity of our proteomics findings. We derived the quantification of specific proteins from the TMT-labeling experiment (2). Only fibrinogen was sourced from HPLs before depletion in TMT-labeling experiment (1) because MARS-hu14 depletion purposefully affects this protein. FGB appeared in larger proportions in FTPL and PPL. Its levels reduced sharply after plasma was taken out of FTPL and after PPL was heat-treated. CD44 was primarily found in HPLs suspended in plasma and saw a marked decrease in PPL, almost to negligible levels. Some CD44 was detectable in HPPL and filtered HPPLs. The group comprising CASP3, SOD1, CD42b, and CD62P had their most pronounced presence in PPL and its derivatives. NSE and HSP70 were mostly seen in PPL alone, a fading exclusive to the western blot analysis. GAPDH consistently appeared in PPL and its related forms, especially prominent in PPL and HPPL. β-actin was not quantified in TMT-proteomics (2) but was measured in TMT-proteomics (1) for both original- and depleted-HPLs. PPL showed the highest β-actin levels, followed by HPPL. In conclusion, the western blot analysis not only reinforced our confidence in the proteomics data but also deepened our understanding of how different processing steps can alter the protein makeup of HPLs.

### D. Preclinical studies of intranasal HPL in mice

#### 1. Implications for brain administration and neuroregenerative potential

**FIG. 7.**
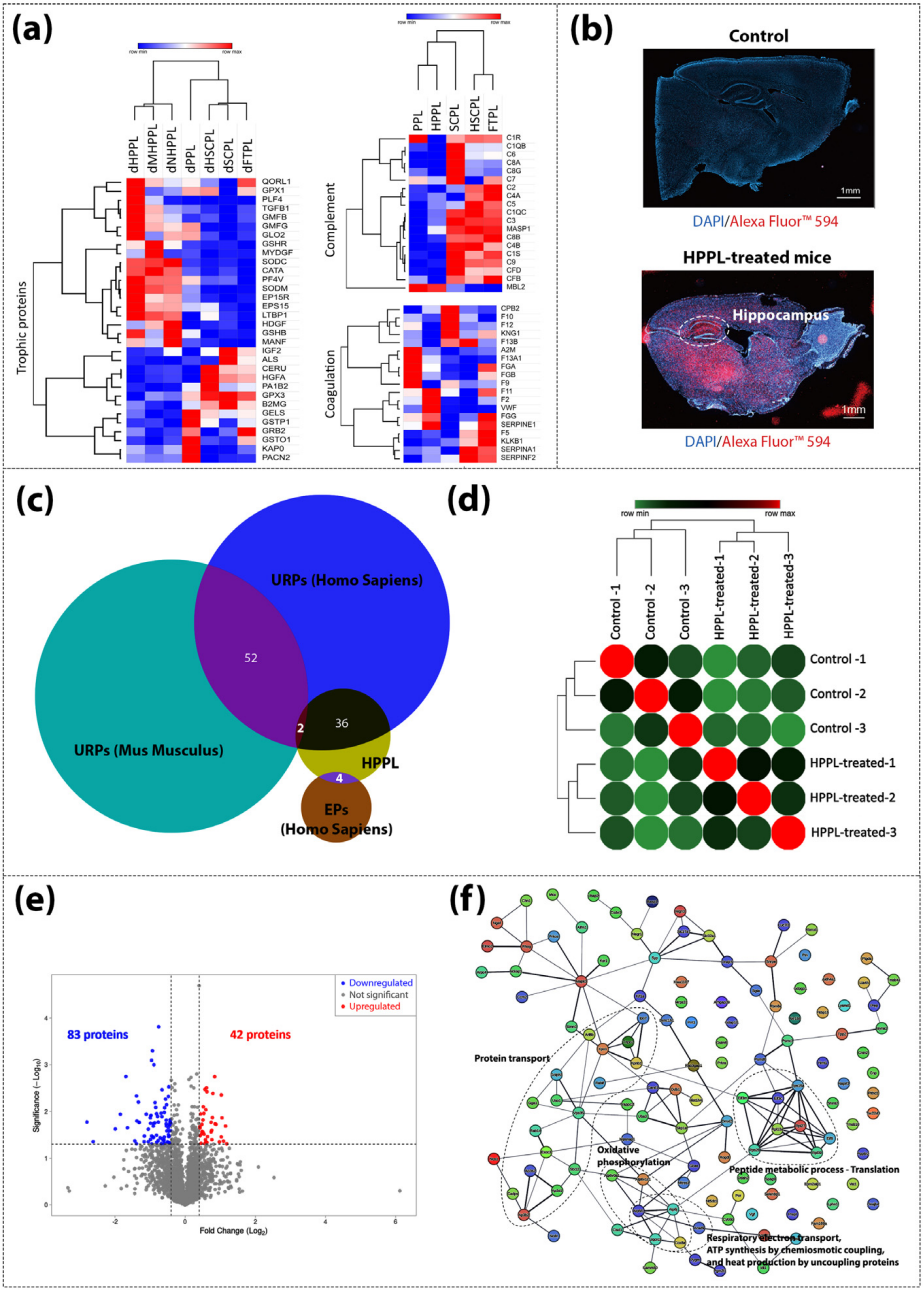
(a) Heatmap of quantification of trophic proteins found in depleted human platelet lysate (HPL) proteomes of the TMT-based experiment (2) and positive regulatory proteins involved in complement and coagulation cascades found in original HPL proteomes of the TMT-based experiment (1); (b) fluorescent labeling of HPPL in sagittal cross section mice brain of control and HPPL-treated group; (c) Venn diagram of exclusive proteins (EPs) identified using the Homo sapiens database and up-regulated proteins (URPs) identified using both the Homo sapiens and Mus musculus databases in HPPL-treated mice group; (d) correlation heatmap between mice hippocampus proteomes; (e) volcano plot of differential-expressed proteins (DEPs) in HPPL-treated mice group; and (f) protein–protein interaction analysis of DEPs.

Platelets contain various trophic factors categorized as cytokines and growth factors in the IPA-general function [Fig. 2(b)] and antioxidants. However, not all blood proteins, such as pro-coagulant and immune-response-associated plasma and platelet proteins, are suitable for brain administration since they could promote thrombus formation or neuroinflammation. Therefore, we assessed two groups of proteins [Fig. 7(a), list of these proteins is supplied in supplementary material 3, Table S5]: trophic factors (including cytokines and growth factors), antioxidants, and neurotrophic factors, which can exert neuroprotective effects on one side, and potentially detrimental complement and coagulation-associated proteins, whose regulatory roles can be found in the Kyoto Encyclopedia of Genes and Genomes—complement and coagulation (pathway mapping is in Fig. S6, supplementary material 2). The quantitative proteomics of complement and coagulation-associated proteins was retrieved from HPLs before depletion in TMT-labeling experiment (1) since most of the proteins involved in this cascade are mainly from plasma and as depletion can affect their relative abundances. In comparisons of dHPLs concerning trophic factors, consistent with our previous studies,^18^ dHPPLs demonstrated a higher relative concentration of these factors. Conversely, in comparisons of HPLs focusing on detrimental proteins, FTPL, SCPL, and HSCPL exhibited a greater abundance of complement cascade proteins and procoagulant proteins. Consequently, HPPL and filtered HPPLs appear better suited for brain administration and neuroregenerative use, given their relative higher proportion. of neurotrophic factors and lower presence of proteins potentially harmful to the CNS. Therefore, subsequently, to further unveil the value of proteomics, we administered HPPL intranasally to mice to study brain diffusion and investigate its effects on the CNS, specifically the hippocampal region which is essential to memory and cognition.

#### 2. Identification of intranasally administered HPPL in hippocampus

We first found that fluorescently labeled HPPL could diffuse into the brain, showing an obvious presence in the hippocampus 7 h post intranasal administration [Fig. 7(b)]. We then used proteomics to confirm the ability of HPPL to reach the hippocampus. The hippocampal tissue of mice that received intranasal HPPL was analyzed using proteomics, employing both Homo sapiens and Mus musculus databases (supplementary material 1, Tables S4 and S5). Up-regulated proteins (URPs), quantified in both databases, and exclusive proteins (EPs) identified using the Homo sapiens database were compared with the top abundant proteins in HPPL. This comparison helped identify proteins from HPPL that, upon reaching the hippocampus, caused accumulation (increased presence) and uniquely presented these proteins in HPPL-treated mice, respectively [Fig. 7(c)]. The proteomics data suggested that HPPL could diffuse to the hippocampus, as indicated in particular, by the detection of two proteins with increased expression and four proteins uniquely present in HPPL-treated mice (Table II).

**TABLE II.** List of detectable HPPL proteins identified as diffusing or accumulating in the hippocampus region of mice upon intranasal administration.^a^

Accession	Description
Highly expressed proteins in HPPL-treated mice	
P55072	Transitional endoplasmic reticulum ATPase OS = Homo sapiens
P62258	14-3-3 protein epsilon OS=Homo sapiens
Unique identifiable proteins in HPPL-treated mice	
P02790	Hemopexin OS = Homo sapiens
P04040	Catalase OS = Homo sapiens
P01859	Immunoglobulin heavy constant gamma 2 OS = Homo sapiens
P02787	Serotransferrin OS = Homo sapiens

^a^ OS: Organism species.

### C. Impact of HPPL on hippocampus

A notable effect of HPPL on the proteome of the mice hippocampus was observed, distinguishing the HPPL-treated mice from the controls with 125 differentially expressed proteins (DEPs) [Figs. 7(d) and 7(e)]. A detailed examination of the processes involving these DEPs highlighted various protein–protein interactions. Specifically, these interactions were linked to processes, such as protein transport, oxidative phosphorylation, peptide metabolism, translation and respiratory electron transport, ATP generation through chemiosmotic coupling, and heat production by uncoupling proteins [Fig. 7(f)].

## III. DISCUSSION

Platelets, generated from megakaryocytes (MKs), are essential for hemostasis^33^ and increasingly acknowledged in tissue repair and healing.^34,35^ The significance of platelets in regenerative medicine is underscored by the development of diverse HPL biomaterials. These biomaterials, depending on their production processes, can exhibit varying protein compositions.^23,36^ While several studies have examined the proteomics of platelet-based products,^37–39^ our research stands in delineating the protein content of HPLs following varied processing steps. Specifically, besides lysing platelets contained in plasma to generate FTPL, we aimed to partially remove plasma proteins to obtain SCPL and centrifuge to obtain only platelets to generate PPL. We applied heat treatment of SCPL and PPL to produce HSCPL and HPPL, respectively. Additionally, HPPL underwent two filtration processes: 0.2 and 0.1-*μ*m microfiltration to obtain MHPPL and 19-nm nanofiltration to produce NHPPL. We rigorously characterized the protein content of all HPLs using methods like BCA, label-free proteomics, TMT-labeling proteomics, and western blot.

We found that each processing phase influenced the protein content in HPLs. Distinctively, we observed different protein contents based on the removal of plasma, heat processing, and nanofiltration methods. Particularly noteworthy is our finding that heating had a profound effect on PPL and its derivatives, most likely due to a deficit of proteins exhibiting some degree of heat-stability, e.g., immunoglobulin, albumin, keratin, and heat-shock protein (visual representation of the abundances of heat-stable proteins are in Fig. S7, supplementary material 2). The nanofiltration process used for virus removal in HPPL did not significantly reduce the total protein content.

Using state-of-the-art LC-MS/MS-based proteomics, our dual approach combining label-free proteomics and TMT-labeled proteomics offers a comprehensive insight into HPL proteomes. Although the platelet proteome has already been investigated,^31,40–42^ the nuanced distinctions in the proteomes of HPL for regenerative medicine have remained unexplored. Comparing our findings with an established platelet protein database by Huang *et al.*,^31^ our research confirms their results and introduces previously unidentified proteins. Most of these proteins comprised cytoplasmic proteins with catalytic activity and are associated with vital biological processes like hemostasis, and immune responses, the primary functions of platelets,^33,43^ and are linked with exosomes, suggesting their critical role in cell-to-cell communication and therapeutic potentials. This observation aligns well with growing evidence of the presence of “exosomes”—or extracellular vesicles (EVs)—in HPLs, as well as their functional role in various biological processes.^44–48^ Platelet EVs are crucial in cell-to-cell communication and may contribute to the functional activity of some HPLs.^49–51^

Our label-free approach unveiled a wider range of proteomes. We observed that the proteins identified in FTPL, SCPL, and HSCPL, were nearly half of those from PPL and its derivatives even though the former had higher total protein contents. Upon examining the top 100 abundant proteins in these HPLs (supplementary material 3, Table S6), gauging them based on the peak area of detected peptides for label-free relative protein quantification,^52,53^ we noticed that 14 prevalent plasma proteins^54^ appeared dominantly across almost all HPLs. Their high abundances could obscure less prevalent proteins and reduce the analysis resolution in LC-MS/MS.^55–57^ Label-free proteomics, although valuable for broad proteome coverage, has limited robustness for quantification and reproducibility compared to labeled methods.^58–61^ To counteract this, we adopted two strategies: first, we utilized immunoaffinity depletion to reduce the abundance of these 14 dominant proteins across all HPLs,^55,62^ enhancing our proteomic analysis resolution, and, second, we incorporated the TMT-labeling approach, a more accurate quantification method, to facilitate a deeper proteomic investigation.

TMT-labeled proteomics was conducted in two separate sets due to limited number of available labels (i.e., 10 plex^32,63^ for a single a single LC-MS/MS run). The first set (TMT-proteomics (1) included paired HPLs both before and after depletion, encompassing the original and immuno-depleted variants of FTPL, SCPL, HSCPL, PPL, and HPPL. The second batch (TMT-proteomics (2) focused solely on immunodepleted HPLs, covering FTPL, SCPL, HSCPL, PPL, HPPL, MHPPL, and NHPPL. PCA was utilized to represent the proteomes of each HPL within a 3D plot. Notably, the consistency of immuno-depleted proteomes was evident by the identical groupings observed across both labeling studies, signifying uniform depletion across all HPLs. Within TMT-proteomics (1), the proteome coverage of dHPLs was marginally greater than that of their original HPLs. Yet, differences in protein abundances pre- and post-depletion were apparent from the PCA results. The subtle rise in protein counts in dHPLs aligns with prior studies,^37,64^ which demonstrated the efficacy of immunodepleting in unveiling platelet proteomes,^55,61^ enabling more precise quantification of low-abundance proteins. Furthermore, we detected a higher protein count in plasma-suspended dHPLs, roughly double the proteins found through label-free methods (Fig. S7, supplementary material 2). This result was anticipated since FTPL underwent no additional processing steps, preserving the complete protein composition of both platelets and plasma. Owing to these insights, the dHPLs batch (TMT-proteomics 2) was chosen for an extensive analysis of protein compositions and functionality. This selection also aimed to shed light on the implications of varying processing methods on HPL.

In the dHPLs set (TMT-proteomics 2), the abundances of each identified protein present were depicted using a heatmap. Through hierarchical clustering, distinct protein profiles for each HPL type became evident. Each group of particularly abundant proteins of each HPLs was annotated to identify the biological processes in which each HPL type plays an important role. In the case of PPL, proteins that were most abundant were chiefly linked to processes like protein folding, intracellular transport, and cytoskeletal organization. These processes are all pivotal in platelet activation where the interaction and signaling between proteins is key, leading to shape changes in platelets and their cohesiveness in response to external stimuli.^37,65^ Moreover, this group of proteins may also contribute to self-organization leading to potential regeneration in tissues.^66,67^ Both HPPL and the filtered HPPLs clustered together, indicative of their similar protein compositions, reflecting protein catabolism. This category encompasses proenzymes and enzymes located within platelets and contributes to hemostasis.^68,69^ Moreover, catabolic proteins might assist in the restoration of oxidized proteins, preserving their functions, and enhancing tissue repair. Such a protective mechanism could be advantageous in the treatment of neurological disorders tied to oxidative stress.^70^

Neurotrophic factors, cytokines, and antioxidants, found in platelets can exert beneficial effects to stimulate neuronal regeneration.^21,71,72^ However, not all blood-derived components are safe for brain delivery. Some proteins like those related to coagulation (e.g., FGB, coagulation factors, and thrombin) and the immune response (e.g., complement factors) may damage the brain.^73–76^ In HPPLs, beneficial proteins were found to be in high proportion (e.g., PF4, TGF, myeloid derived growth factor—MYDGF), whereas in FTPL, SCPL, and HSCPL, proteins potentially unsafe for the CNS were found to be prominent. In the context of neuroregenerative applications, this demonstrates that HPPL and derivatives have a better potential to be a safe and effective treatment, confirming preclinical observations.^14,18,19,77^

Furthermore, the knowledge gained from HPPL proteomics proved to be valuable in assessing its diffusion to the hippocampus following i.n. administration in mice. The hippocampus was selected as a distant brain region easily differentiated from olfactory structures where proteins delivered intranasally might be detected readily. In addition, its role in memory functions and its impairments in various neurological disorders, including Alzheimer's disease,^16,21,76,78^ makes it a focal point of interest for clinical translation. Our fluorescent imaging of labeled HPPL also illustrated accumulation in the hippocampal region. It is important to acknowledge that using proteomics in pre-clinical rodent models has inherent limitations, particularly due to the structural similarities between human and mouse proteins, which pose challenges in fully distinguishing between the two species. Therefore, to study protein diffusion into the hippocampus, two databases were utilized for peptide sequence matching in the proteomics analysis of extracted hippocampal tissue. Complementing this with proteomics suggested the presence of HPPL proteins as valosin-containing protein, 14-3-3 epsilon protein, hemopexin, catalase, immunoglobulin, and serotransferrin in the mice hippocampus. ELISA could also potentially detect subtle increases in HPPL proteins in various brain areas, as previously evidenced by tracing PF4 in mice.^14^ Proteomics analysis has revealed that i.n. HPPL administration significantly impacts transport, metabolic, and translation processes. These processes are crucial for neurogenesis, as they provide the dynamic cellular activities for the generation, differentiation, and integration of new neurons. This suggests that HPPL could offer therapeutic strategies for promoting neural repair and regeneration. In light of these findings, we have initiated studies to investigate the potential neurogenesis effects of HPPL treatment in the dentate gyrus (DG) region of the hippocampus. Our preliminary data (Nyam-Erdene *et al.*, unpublished) indicate that both 3-day and 28-day i.n. administration of HPPL can stimulate neuronal proliferation and differentiation in the DG, as evidenced by increased Neu-N expression. These data corroborate previous research by our team (Nebie *et al.*, Brain),^17^ where HPPL was shown to improve memory functions in a traumatic brain injury (TBI) model. Together, these results suggest a positive effect of HPPL on neurogenesis and memory, underscoring its potential as a therapeutic agent for enhancing brain health and function. On-going studies aim to further elucidate the mechanisms behind these effects and to explore the translational applications of these findings for treating neurological disorders and injuries.

HPLs suspended in plasma (i.e., FTPL, SCPL, and HSCPL) showed unique protein expression patterns tied to hemostasis, complement activation, and innate immune responses. This suggests they might interact with the immune system.^79^ Moreover, HSCPL and FTPL displayed proteins related to retina homeostasis and cell adhesion. Cell adhesion is crucial for controlling cells, their interactions, and communication with the extracellular matrix (ECM), all of which are important for healing wounds.^80^ Given its protein content, HSCPL and FTPL could be beneficial for protein adhesion and treatments involving ocular, skin, and joint repair.^11,28,81,82^ In previous studies, we and others have indeed observed the benefit of using the heat-treated HSCPL for the expansion of corneal endothelial cells^28,82^ Also, FTPL is a prototype of HPLs found to be valuable for the treatment of skin ulcers and of osteoarthritis.^3^

To generate various HPL types tailored for different clinical needs, we employed specific processing steps. These steps led to variations in protein number and abundance (refer to Fig. S4, supplementary material 2). While a comprehensive breakdown of all protein changes due to these steps is beyond this study's scope, we used IPA to depict shifts in protein abundance through associated metabolic pathways. Through clustering, we noticed treatment effects splitting into two main groups: those for obtaining SCPL and its derivatives and those for PPL and its derivatives. Intriguingly, there was opposing regulation of some canonical pathways between these groups. The process of seroconversion to generate SCPL by activating platelets using calcium salt affected extracellular platelet receptors.^83^ This activation triggered an acute endocytic response to inflammation, upregulating related pathways like integrin, actin, and cell adherens signaling. Conversely, plasma removal in PPL production and the microfiltration in MHPPL seems to turn-down these signals, possibly due to LXR/FXR signaling activation. This signaling can hinder platelet activation^37^ and/or reduce platelet intracellular protein content. Heat treatment applied to both SCPL and PPL, along with MHPPL nanofiltration, caused only subtle changes in canonical pathways. Notably, heat treatment decreased complement system signaling in SCPL. Heating in PPL and HPPL microfiltration, it was mainly platelet intercellular signaling-related and coagulation proteins that saw alterations. Our rigorous IPA-based findings underline the significant role of processing steps in shaping the proteomic profile of HPLs. These changes in protein levels offer crucial insight into their functional implications.

We demonstrated the agreement between proteomics-based quantification and semiquantitative immunoblotting for several proteins. These included plasma proteins like FGB and CD44,^84,85^ platelet intracellular proteins, such as CASP3, SOD1, NSE, HSP70, GADPH, and β-actin,^86–89^ and proteins on the surface of platelets or EVs surface like CD42b, and CD62P.^90^ PPL and FTPL exhibited the highest relative abundances. However, activation with calcium salt and heat treatment led to a reduction in FGB quantity in these samples. When the plasma component was removed, CD44 expression significantly decreased, but its relative abundance increased in subsequent derivatives. PPL and its derivatives were rich in intracellular proteins, as well as CD42b and CD62P, which are markers for non-activated and activated platelets, respectively. This indicates a high platelet content. Notably, NSE and HSP70 showed reduced abundance after heat treatment, while GAPDH and β-actin levels dropped during the filtration process.

This study provides a comprehensive proteomic profile of HPLs, serving as a foundation for future research. It elucidates how processing methods generally influence the composition of HPLs. While the plasma component retains proteins linked to complement pathways and wound healing adhesion, calcium-mediated activation boosts integrin and actin-related pathways. However, heating reduces complement proteins. Hence, applying both activation and heating on plasma-suspended platelets yields a set of proteins which are found to be especially beneficial for retinal homeostasis. Upon thorough removal of plasma, there is a reduced potential of platelet activation, with predominantly intracellular proteins being affected by the treatments, resulting in the emergence of distinct protein cohorts. Notably, PPL is rich in chaperones and transport proteins, while HPPL and its derivatives have a higher concentration of catabolic proteins. This suggests their regenerative potential. Moreover, with HPPL and its derivatives being rich in trophic factors and heating, combined with microfiltration helping minimize coagulation proteins, they emerge as prime candidates for assisting neuroregeneration due to their efficacy and safety profile. Nanofiltration impact is not dramatic, making it a valuable process to ensure HPPL viral safety without significantly altering protein composition.^19^ Even though our data show significant differences in the proteomes of HPLs depending on their preparation methods, we must acknowledge that variations within the same type of HPL could be linked to the methods how the PCs are collected, the proteomic methods used, and the size of the HPL pool. These factors need to be considered to contextualize the extent of the observed differences among different HPLs and within each HPL category. To further evidence the presence of HPPL in the hippocampus, proteomic analysis serves as an initial step, suggesting the presence of specific HPPL proteins within this brain region. Thus, the observed differences between treated and untreated groups are thought to result from the HPPL treatment. However, the definitive presence of these proteins warrants confirmation in future studies through molecular techniques.

## IV. CONCLUSIONS

Our study identified a total of 1689 proteins in platelet lysate biomaterials using label-free and TMT-labeled proteomics. These proteins were associated with platelets and exhibited catalytic activities, predominantly originating from the cytoplasm. The proteomes of different HPLs were characterized by distinct protein profiles, with PPL enriched in proteins related to platelet shape changes and HPPL and its derivatives rich in proteins involved in protein catabolism. HPLs derived from platelets suspended in plasma displayed proteins associated with complement interactions, with FTPL and HSCPL containing specific classes of proteins with potential benefits for ocular and wound healing. Our findings support the therapeutic potential of HPPL and filtered HPPLs, which exhibited relatively high levels of trophic proteins and low proportions of complement proteins, for neuroregenerative medicine. Proteomics and fluorescence imaging also proved to be useful—albeit with some limitations—to study the diffusion of i.n. HPPL to mouse hippocampus and to observe effects on the biological pathways. This study provides valuable insight into the protein compositions of HPLs, offering valuable implications for regenerative medicine, and strongly supporting the need for developing tailor-made HPLs for use in cell therapy and regenerative medicine.

## V. METHODS

### A. PC collection and HPLs preparation

**FIG. 8.**
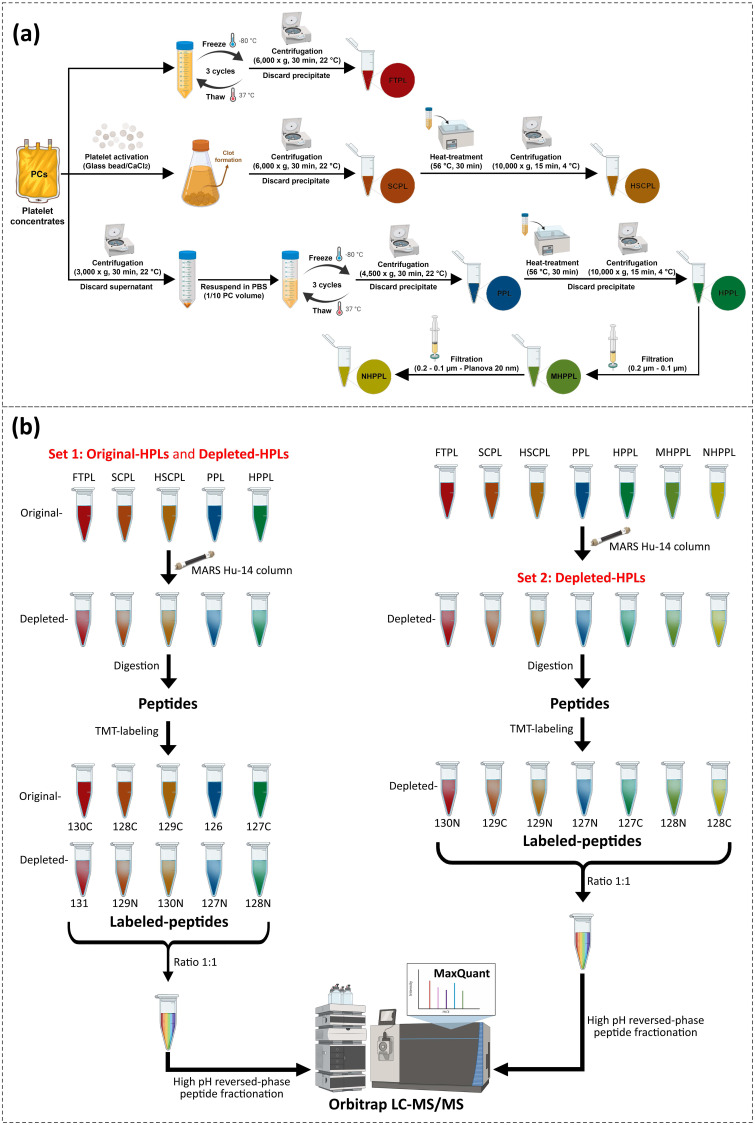
(a) Human platelet lysates (HPLs) preparation scheme and (b) tandem mass tag (TMT)-based LC-MS/MS scheme for original human platelet lysates (HPLs) and depleted-HPLs. Abbreviations: Freeze-thawed platelet lysate (FTPL), serum-converted platelet lysate (SCPL), heat-treated SCPL (HSCPL), platelet pellet lysate (PPL), heat-treated PPL (HPPL), filtered HPPL: 0.2–0.1 *μ*m (MHPPL), and Planova 20 N (NHPPL).

The detailed description of PC collection and the processing steps for each HPL are supplied in the supplementary material.^14,18,19,23^ The HPLs preparation scheme is shown in Fig. 8(a). The study was approved by the Institutional Review Board of Taipei Medical University (TMU-JIRB N201802052) and the Taiwan Blood Services Foundation (Taipei, Taiwan). Briefly, the PCs were processed in three different manners for providing seven individual types of HPLs: freeze-thawed platelet lysate (FTPL), serum-converted platelet lysate (SCPL), heat-treated SCPL (HSCPL), platelet pellet lysate (PPL), heat-treated PPL (HPPL), filtered HPPL: 0.2–0.1 *μ*m (MHPPL), and Planova 20 N (NHPPL). FTPL was prepared by freezing and thawing PC (−80 ± 1/37 ± 1 °C) three times to disrupt platelet membranes and release platelet contents, followed by centrifugation.^23^ Calcium chloride was added to PCs to activate and degranulate platelets to prepare SCPL. The fibrin clot and glass beads were then removed by centrifugation of the suspension. SCPL was heated to 56 ± 1 °C for 30 min and then immediately cooled down on ice for 5 min, followed by centrifugation to remove any precipitate to prepare SCPL.^4^ To prepare PPL, PCs were centrifuged to pelletize platelets that were recovered and resuspended in PBS using a volume of 10% of that of the initial PCs. The re-suspended pellet was subjected to three freeze and thaw cycles, and centrifuged. HPPL was produced by heat-treatment of PPL, followed by centrifugation to remove any precipitate.^18,23^ HPPL was pre-filtered on 0.22-*μ*m PN 4612 and 0.1-*μ*m PN 4611 filters (Pall Life Science, Ann Arbor, MI, USA) to form MHPPL. MHPPL were then filtered through 0.001-m^2^ planova-20N (19-nm pore size; Asahi Kasei, Tokyo, Japan), a filter dedicated to the removal of blood-borne viruses, to form NHPPL.^19^ To minimize individual differences in the proteome of platelet concentrates, we pooled the HPLs from three different donors.

### B. HPL proteomics analysis

The total protein content of HPLs was assessed by a BCA assay (BCA Protein Assay Kit; Thermo Scientific™ Pierce™, Rockford, IL, USA). Label-free LC-MS/MS analysis was performed on all HPLs as outlined in previous publications.^18,19,44^ We performed TMT-labeled proteomics in two sets due to limited TMT labels (i.e., TMT10plex™,^32,63,91^ following the general scheme illustrated in Fig. 8(b). The first set included pairs of HPLs before immunodepletion (FTPL, SCPL, HSCPL, PPL, and HPPL) and after immunodepletion (dFTPL, dSCPL, dHSCPL, dPPL, and dHPPL), while the second set comprised all HPLs after immunodepletion (dFTPL, dSCPL, dHSCPL, dPPL, dHPPL, dMHPPL, and dNHPPL), allowing for a comprehensive analysis of both plasma and platelet composition. Digested peptides were labeled with TMT-labels and post-fractionated using a high-pH, reverse-phase peptide fractionation kit. Quantitative proteomics analysis used label-free and TMT-labeled peptides, with LC-MS/MS performed on an Orbitrap Fusion Lumos Tribrid Mass Spectrometer (Thermo Fisher Scientific, San Jose, CA, USA). Additional information on the proteomics methodology can be found in supplementary material 2.

### C. Mice hippocampus proteomics analysis after HPPL administration

Animal experiments were performed following protocols approved by the Institutional Animal Care and Use Committees of the Taipei Medical University (Protocol No. LAC-2019-0020). Mice (8–10 weeks old) received intranasal administration of 60 *μ*l HPPL daily, at a dose of 3 *μ*l per nostril, alternating between nostrils, for 7 days. Mice were then sacrificed by cervical dislocation. After washing the brains with cold-PBS, hippocampus was harvested, homogenized, checked for total protein content by BCA and submitted to sample preparation for label-free LC-MS/MS as described in supplementary material 2. PBS was used as negative control.

### D. Mice brain diffusion study of HPPL

HPPL was labeled with Alexa Fluor 594 dye (Thermo Fisher Scientific, San Jose, CA, USA) following the supplier instructions. A total volume of 60 *μ*l of fluorescently labeled HPPL was administered intranasally. The mice were anesthetized 7 h after the final HPPL administration using Zoletil-50 (66F4, Virbac, France) and Rompun (PP1523, Bayer, Swit) and perfused with 0.9% cold NaCl. Brains were fixed in 4% PFA and transferred in cryoprotective solution. Sagittal cryo-sections (30 *μ*m) of the harvested brains were obtained for observation by a fluorescent slide scanner.

### E. Bioinformatics analysis

Proteins with a ratio of >1.3 or <0.769 were considered significantly varied in abundance.^91^ The DAVID (Database for Annotation, Visualization and Integrated Discovery) functional annotation tool (https://david.ncifcrf.gov/) and Ingenuity pathway analysis (IPA; Qiagen, Redwood City, CA, USA) software were used for the functionality and pathway enrichment analyses. Venn diagrams were drawn using the Python matplotlib-venn package. A principal component analysis (PCA) was applied using the open-source sklearn.decomposition of three-dimensional configuration. Morpheus (Broad Institute, Cambridge, MA, USA; https://software.broad-institute.org/morpheus) was used to construct heatmaps. Protein–protein network construction was performed by STRING (https://string-db.org), followed by Cytoscape software.^92^ Both Mus Musculus database and Homo Sapiens database were utilized in the label-free MS/MS data acquisition to identify the presence of HPPL proteins in mice hippocampus. The approach followed and illustrated is in Fig. S1, supplementary material 2.

### F. Western blotting

HPLs were separated on SDS gel and blotted onto polyvinylidene fluoride (PVDF) membrane using standard protocols. Sample processing, analysis, and reagents used are described in supplementary material 2.

### G. Statistical analysis

Statistical analyses were performed using GraphPad Prism software version 6.0 (La Jolla, CA, USA). The data are presented as the mean ± standard deviation (SD). A one-way analysis of variance (ANOVA) followed by Dunnett's multiple-comparisons (DMC) test was performed for comparison, and differences were considered significant at *p* < 0.05.

## SUPPLEMENTARY MATERIAL

See the supplementary material for details regarding supplementary material 1: Table S1—list of all proteins identified following quantitative label-free LC-MS/MS, Table S2—list of all proteins identified following TMT-labeling experiment (1) of HPLs pairs before and after depletion, Table S3—list of all proteins identified following TMT-labeling experiment (2) of the seven depleted-HPLs, Table S4—list of all proteins identified following Quantitative label-free LC-MS/MS in mice hippocampus using Mus Musculus database, Table S5—list of all proteins identified following quantitative label-free LC-MS/MS in mice hippocampus using Homo sapiens database; supplementary material 2: part 1: Materials and methods: PC collection, preparation of different HPLs, sample preparation for label-free LC-MS/MS, sample preparation for TMT-labeling LC-MS/MS, LC-MS/MS data acquisition, MS data processing, proteome identification and quantitation, bioinformatics analysis, and Western blotting; Part 2: Results: Fig. S2—Venn diagram of proteins identified in the HPLs compared with proteins annotated in the Exocarta and Vesiclepedia databases, Fig. S3—number of quantified proteins in TMT-labeling proteomics of HPLs before and after immunodepleting of 14 high-abundance plasma proteins, Fig. S4—Pearson correlation heatmap of HPLs proteomes showing groups with high correlation were HPPL-MHPPL-NHPPL, and FTPL-SCPL-HSCPL, Fig. S5—the amount of proteins varied in abundance significantly when comparing before and after being process steps, Fig. S6—heatmap of various heat-stable proteins in plasma annotated in TMT-labeling experiment (2) of depleted-HPLs, Fig. S7—number of identified proteins in label-free experiment of original-HPLs and in TMT-labeling experiment (2) of depletion-HPLs, Fig. S8—KEGG pathways of proteins involved in complement and coagulation cascades; and supplementary material 3: Table S1—list of previously unidentified proteins in the platelet proteome, Table S2—list of exclusive proteins between the original- and depleted-HPLs, Table S3—list of common and exclusive proteins between the seven depleted-HPLs, Table S4—list of highly abundant proteins specific to HPLs, Table S5—list of heat-stable proteins annotated in TMT-labeling experiment (2) of the heat depletion-HPLs, Table S6—list of 32 trophic proteins and 38 proteins from complement and coagulation cascades found in TMT-based approaches.

## Data Availability

The data that support the findings of this study are available within the article and its supplementary material.
